# Supplementary material for: Isomeric O-methyl cannabidiolquinones with dual BACH1/NRF2 activity
Source: Redox Biol. 2020 Aug 22;37:101689. doi: 10.1016/j.redox.2020.101689 (PMC7476313; doi:10.1016/j.redox.2020.101689)
Supplement: Multimedia component 1 [file mmc1.docx]

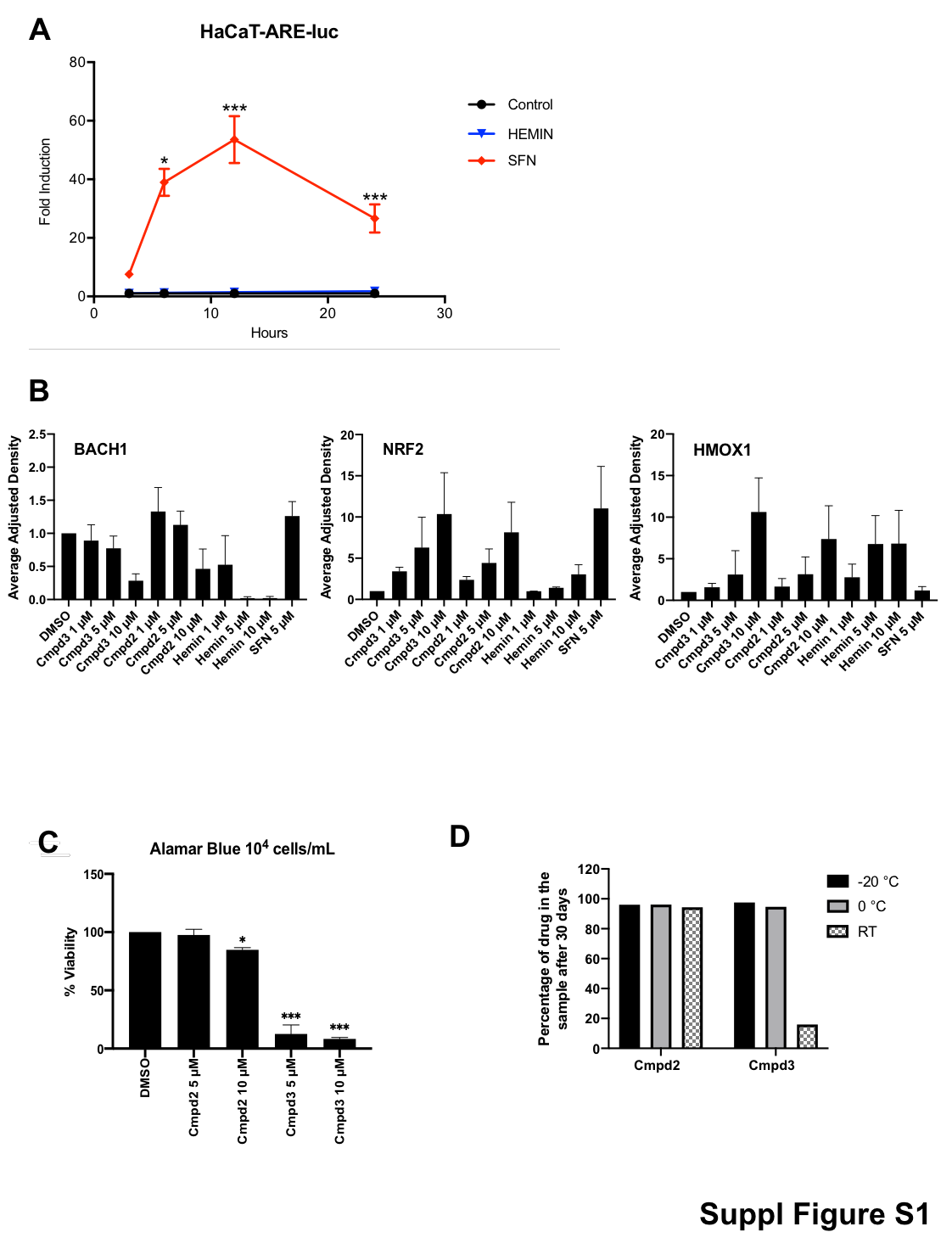


** Suppl. Figure S1. A)** HaCaT-ARE-Luc cells were treated with either DMSO, SFN (5 µM) or hemin (5 µM) for different times as indicated. Luciferase activity was measured in the cell lysates and expressed as RLU (x 10^4^). Data represent means ± SD (n = 3) and are expressed relative to untreated cells. *P ≤ 0.05, **P≤ 0.01, ***P ≤ 0.001. **B)** BACH1, NRF2 and HMOX1 protein quantification against the loading control for the representative blot in Fig. 1D. Data represent means ± SD (n = 3) and are expressed relative to the DMSO treated samples. **C)** HaCaT cells were seeded in 96-well plates, and treated with DMSO or increasing concentrations of compound 3 or compound 2. 16 hours later, Alamar Blue was added to the wells and the cells were incubated for 4 hours at 37 °C. The fluorescence was measured using a plate reader and viability was calculated relative to the untreated control (n=3). *P ≤ 0.05, **P≤ 0.01, ***P ≤ 0.001. **D)** Drug samples were stored under not controlled conditions at room temperature (20-30 °C), 0 °C or -20 °C. The samples were retested after 30 days by HPLC. Percentages are expressed relative to a reference sample. **E)** Hepa-1c1c7 cells were exposed to either DMSO or increasing concentrations of compound 2, hemin or SFN for 3 hours. Cells were lysed and NRF2, HMOX1 and Actin protein levels were analyzed by Western Blot. Upper panel is a representative western blot and bottom panels show the quantification of NRF2 and HMOX1 protein levels against the loading control. Data represent means ± SD (n = 3) and are expressed relative to the DMSO treated samples. **F)** Hepa1c1c7 cells were treated with eight different concentrations of compound 2 in eight replicates. After 48 hours cells were lysed and the specific activity of NQO1 was analysed. The average of two independent experiments is shown. **G)** The mRNA levels of the indicated genes were were quantified by real-time PCR in WT, BACH1-KO (upper panel) or NRF2-KO (lower panel) HaCaT cells. The data were normalised using *HPRT1* as an internal control. Data represent means ± SD (n = 3) and are expressed relative to the WT sample. * P≤0.05, **P≤ 0.01, ***P ≤ 0.001. **H)** HaCaT cells were treated with either DMSO, compound 2 (5 µM), Hemin (5 µM) or SFN (5 µM) for 8 hours. The mRNA levels of the indicated genes were quantified by real-time PCR and the data were normalised using *HPRT1* as an internal control. Data represent means ± SD (n = 3) and are expressed relative to the DMSO sample. * P≤0.05, **P≤ 0.01, ***P ≤ 0.001.

**Suppl. Figure S2. A)** HaCaT WT and BACH1-KO cells were harvested and mRNA levels of *HMOX1* were quantified by real-time PCR. The data were normalized using HPRT1 as an internal control. Data represent means ± SD (n=3) and are expressed relative to WT sample. **P≤ 0.01 **B)** HaCaT WT and BACH1-KO cells were harvest and lysed. BACH1, HMOX1 and Actin protein levels were analysed by Western Blot. **C)** HaCaT WT and NRF2-KO cells were incubated with either DMSO or SFN (5 µM) for 3 hours. Cells were lysated and NRF2 and Actin protein levels were analysed as previously described. **D)** HaCaT WT and NRF2-KO cells were collected and lysed. mRNA levels for *NQO1*, *AKR1B10* and *HMOX1* were quantified using real-time PCR (n=3). ns P ≥ 0.05, ***P ≤ 0.001. **E)** HaCaT NRF2-KO cells and HaCaT NRF2-KO/BACH1-KO cells were harvested and lysed. BACH1, NRF2, HMOX1 and Actin protein levels were analysed by Western Blot.

**Suppl. Figure S3. A)** Quantification of BACH1 and HMOX1 protein levels against the loading control from Fig. 2E. Data represent means ± SD (n = 2) and are expressed relative to the siControl DMSO sample. **B)** A549 cells were incubated with DMSO, SFN (5 µM) or increasing concentrations of compound 2 for 3 hours. Cells were lysed and NRF2 and Actin protein levels were analysed by Western Blot.
